# DNA damage in BALB/c mice infected with *Lacazia loboi* and its relation to nutritional status

**DOI:** 10.1186/s40409-015-0006-y

**Published:** 2015-03-25

**Authors:** Adriana Sierra Assencio Almeida Barbosa, Larissa Ragozo Cardoso de Oliveira, Francilene Capel Tavares, Carlos Roberto Gonçalves de Lima, Suzana Madeira Diório, Sueli Aparecida Calvi, Fátima Regina Vilani-Moreno, Paulo Câmara Marques Pereira

**Affiliations:** Biology Technical Team, Lauro de Souza Lima Institute, Bauru, São Paulo State Brazil; Department of Tropical Diseases and Image Diagnosis, Botucatu Medical School, São Paulo State University (UNESP – Univ Estadual Paulista), Botucatu, São Paulo State Brazil; Microbiology Technical Team, Lauro de Souza Lima Institute, Bauru, São Paulo State Brazil; Equipe Técnica de Biologia, Instituto Lauro de Souza Lima, Rod. Comte. João Ribeiro de Barros, km 225/226, CEP 17.034-971 Bauru, SP Brazil

**Keywords:** Jorge Lobo’ disease, *Lacazia loboi*, Malnutrition, Mice, Genotoxicity, Comet assay

## Abstract

**Background:**

Jorge Lobo’s disease, also known as lacaziosis, is a cutaneous-subcutaneous mycosis with chronic evolution. It is caused by the fungus *Lacazia loboi*. Herein we report a study that relates the genotoxicity caused by *L. loboi* in isogenic mice with nutritional status, through a normal or restricted diet.

**Methods:**

DNA damage was assessed in the peripheral blood by the comet assay (tail intensity).

**Results:**

The results for leukocytes showed increases in the mean tail intensity in mice under dietary restriction, in infected mice under dietary restriction and in infected mice ingesting a normal diet.

**Conclusion:**

These results indicate that dietary restriction and *L. loboi* infection may increase DNA damage levels in mice, as detected by the comet assay.

## Background

 Jorge Lobo’s disease was first described in 1931 by Dr. Jorge de Oliveira Lobo, in Recife, Brazil. It was observed in a 52-year-old male patient who worked on an Amazon rubber-tree plantation and had shown nodules on the lumbosacral and gluteal regions for 19 years [1,2].

The disease, also known as lobomycosis or lacaziosis, is a chronic, granulomatous, cutaneous-subcutaneous, fungal infection caused by the fungus *Lacazia loboi* and characterized by isolated or multiple coalescing lesions. These lesions usually show a keloidal aspect and are located primarily on the auricular pavilion and limbs of patients [3,4]. Since it is not compulsory that cases of Jorge Lobo’s disease be reported, the actual number of patients is unknown; however, this affliction is endemic in the Brazilian Amazon region and has presented fewer cases in other countries [2,5].

 The fungus *L. loboi* has not been grown in artificial culture media so far, and, despite several studies attempting to experimentally reproduce Jorge Lobo’s disease, only a few showed positive results [1,2]. For example, Madeira *et al.* [6] and Belone *et al.* [7] suggested that the BALB/c mouse strain is an excellent model for maintaining *L. loboi* in the laboratory.

 Malnutrition has been reported as the main cause of immunodeficiency, and can lead to serious infections and mortality [8-10]. Several processes may be affected by nutritional state, such as antibody production, microbicidal capacity of phagocytes, and production of pro- and anti-inflammatory mediators [11]. These changes in the immune response may be responsible for an intense inflammatory reaction that can lead to oxidative stress, one of the mechanisms associated with genotoxic changes [8-11].

Several infectious agents have been evaluated as carcinogenic to humans. Most of this cancer burden was related to infection with viruses, and a small portion to bacterial and parasitic infection with human papillomaviruses, hepatitis C virus, *Helicobacter pylori, Schistosoma haemotobium* or *Trypanosoma cruzi* [12-14].

Taking into account the relationship between DNA and carcinogenesis, the single-cell gel electrophoresis (comet) assay is a new, rapid, simple and easily performed biochemical technique for assessing DNA damage in eukaryotic cells [15,16]. The basic principle of the comet assay is the migration of DNA fragments in an agarose matrix during electrophoresis. When observed under a microscope, the cells show a comet appearance, with a head (nuclear region) and a tail containing DNA fragments that have migrated towards the anode [17-19]. The comet assay was chosen to evaluate DNA damage in *L. loboi*-infected animals.

Based on the above considerations, the following two questions should be asked: can lacaziosis induce DNA damage, and does lacaziosis associated with dietary restriction induce or enhance DNA damage in peripheral blood cells from infected BALB/c mice?

## Methods

### Experimental groups

A total of 40 isogenic 12-week-old male BALB/c mice were kept at the Lauro Souza Lima Institute. All animals were housed in plastic cages (five mice per cage) with white wood chips for bedding and access to the commercial food Nuvilab® CR-1 (Nuvital, Brazil), which is specifically made for the feeding of laboratory rodents, and drinking water, under conditions of controlled lighting (12-hour day/12-hour night cycle) and temperature (22 ± 2°C). The animals were distributed into four groups: (G1) ten mice inoculated with the fungus and under dietary restriction, (G2) ten mice not inoculated with the fungus and under dietary restriction, (G3) ten mice inoculated with the fungus and on normal diet and (G4) ten mice not inoculated with the fungus and on normal diet. The research project was approved by the Ethics Committee on Animal Experimentation of Botucatu Medical School, UNESP.

### Fungal suspension

*L. loboi* was obtained from the footpads of BALB/c mice previously inoculated for maintenance of the strain [7]. The animals were sacrificed and their footpads removed and macerated in 0.9% sterile saline. The fungal suspension (pool) obtained was evaluated with respect to the number of fungi whereas viability was determined by vital staining with fluorescein diacetate-ethidium bromide, as described by Vilani-Moreno and Opromolla [20].

### Inoculation

Both hind footpads of the BALB/c mice were intradermally inoculated with 0.03 mL of the fungal suspension containing 1.4 × 10^6^ fungi, with inoculum concentration of 4.8 × 10^7^/mL and viability index of 38%. The molecularity of the strain used in our experiments was characterized according to Vilela *et al.* [21].

### Diet and weight of the animals

Before the start of the study, at the initial moment, all animals were weighed; the animals in groups G1 and G2 were submitted, for 20 days, to a diet restricted by means of a daily offering comprising 80% of the quantities ingested by groups G3 and G4. The dietary restriction was conducted in order to achieve a weight loss of about 20%. Twenty days after receiving a normal diet or dietary restriction, the animals were infected. After the inoculation, the animals of groups G1 and G2 remained on dietary restriction until the final moment when they had to be sacrificed. All groups of animals were weighed monthly. The food and animals were weighed on a digital scale with a precision of 0.25 g to 5000 g (model AS5500C Marconi, Brazil).

### Euthanasia of the animals

Four months after the inoculation – period necessary for the appearance of the macroscopic lesions on the footpads, according to Belone *et al.* [7] – the animals were euthanized using carbon dioxide (CO_2_) after which peripheral blood was collected.

### Comet assay

The protocol used for assessment of DNA damage in peripheral blood was in accordance with Sasaki *et al.* [22] with some modifications. Peripheral blood (10 μL) was mixed with 120 μL of low melting-point agarose (0.5%) at 37°C, and the mixture was added to duplicate slides previously covered with 1.5% regular agarose; a coverslip was placed on top. After the agarose had solidified in a refrigerator, the coverslips were removed and the slides immersed in a lysis solution (2.5 M NaCl, 100 mM EDTA, 10 mM Tris-HCl buffer, pH 10, 1% sodium sarcosinate with 1% Triton X-100, and 10% DMSO) for 1 hour.

Prior to electrophoresis, the slides were washed in PBS, placed in alkaline solution (1.0 mM EDTA, 0.3 M NaOH; pH > 13) for 20 minutes, and then submitted to electrophoresis for 20 minutes at 25 V/cm, 300 mA. After electrophoresis, the slides were neutralized in 0.4 M Tris-HCl buffer (pH 7.5), fixed in absolute ethanol and stored for analysis. All steps were performed under low-lighting conditions. Duplicate slides were stained with SYBR® Gold (1:10.000 – Invitrogen, USA) whereas 100 nucleoids (50 per slide) were examined at 100× magnification, using an immunofluorescence microscope connected to an image analysis system (Comet Assay II, Perceptive Instruments, UK). DNA damage was measured by analyzing the tail intensity (% of migrated DNA) and tail moment [the product of the tail length (DNA migration) and fraction of DNA in the comet tail, i.e. % DNA in the tail] [16]. As the groups showed statistically significant differences among these parameters, we chose tail intensity to present our results.

### Statistical analysis

All data were analyzed with the Genmod procedure in the software SAS for Windows, version 9.2. The gamma distribution was performed for the analysis of the comet assay. The statistical analysis of body weights was performed by the Tukey test. Results were considered significant when p < 0.05.

## Results

Mean body weights during the experimental period are presented in Table 1. Initially, no significant difference in body weight was observed between the study groups. Then, the animals in groups G1 and G2 were submitted to dietary restriction. In these groups, there was a significant weight reduction at the moment of inoculation and at the end of the study, when compared with the initial moment. In groups G3 and G4, a significant weight increase was observed when the moments were compared. The results revealed that the infection by *L. loboi* did not have any influence on the body weight of the mice. Thus, both infected groups (G1, G3) presented results similar to those of their respective controls.

**Table 1:** **Body weights, in grams, of BALB/c mice according to studied groups and specific moments**

**Groups**	**Body weights**					
	**Initial**		**Inoculation**		**Final**	
	$\overline{\mathbf{x}}$	***s*** ^**2**^	$\overline{\mathbf{x}}$	***s*** ^**2**^	$\overline{\mathbf{x}}$	***s*** ^**2**^
**G1**	25.75	1.33 aA	21.37	1.18 bA	21.57	1.09 bA
**G2**	26.13	2.37 aA	21.28	1.89 bA	21.03	2.14 bA
**G3**	26.36	1.71 aA	27.83	1.72 bB	32.47	1.91 cB
**G4**	24.38	0.97 aA	27.63	1.63 bB	32.58	1.63 cB

Initial: before animals were submitted a diet restriction. Inoculation: animals were intradermally inoculated with *L. loboi* (both footpads)*.* Final: four months after the inoculation. G1: mice inoculated with the fungus and under dietary restriction, G2: mice not inoculated with the fungus and under dietary restriction, G3: mice inoculated with the fungus and on normal diet and G4: mice not inoculated with the fungus and on normal diet. $\overline{x}$: means. *s*^2^: standard deviation. Means and standard deviations followed by the same lower-case letter (line) represents no significant difference by the Tukey test at a level of 5%. Means followed by the same capital letter (column) represents no significant difference by the Tukey test at a level of 5%. Initial body weights: G1 = G2 = G3 = G4. Inoculation body weights: G1 and G2 < G3 and G4. Final body weights: G1 and G2 < G3 and G4.

**Figure 1:**
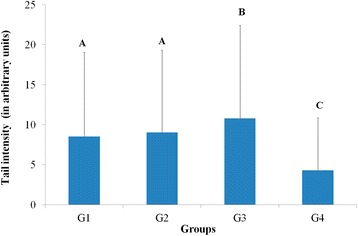
**DNA damage analysis (tail intensity) in peripheral blood through comet assay.** G1: mice inoculated with the fungus and under dietary restriction, G2: mice not inoculated with the fungus and under dietary restriction, G3: mice inoculated with the fungus and on normal diet and G4: mice not inoculated with the fungus and on normal diet. Results are represented as mean and standard deviation. Same capital letter represents no significant difference by the gamma distribution test at a level of 5% (G1 = G2; G3 > G1, G2, G4; G4 < G1, G2, G3).

Figure 1 shows the results of the DNA damage analysis (tail intensity) in peripheral blood, by use of the comet assay. Groups G1, G2 and G3 differed significantly from G4. No difference was observed between the groups with dietary restriction (G1 and G2). Group G3 showed greater DNA damage. Considering that animals in the group G4 (on normal diet) did not present DNA damage, we suggest that the food utilized does not contain a component or additive that causes DNA damage in lymphocytes.

## Discussion

In this study, we investigated whether an infection by *L. loboi* coupled with dietary restriction could induce DNA damage *in vivo*, in peripheral blood from BALB/c mice.

The results of the present work show that cells from infected animals present DNA damage similarly to other studies reporting that microorganisms such as *Trypanosoma cruzi*, *Opisthorchis viverrini*, *Leishmania chagasi*, *Toxoplasma gondii*, and *Mycobacterium tuberculosis* can induce DNA damage [14,17-19,23]. Some studies have demonstrated that certain fungi – such as *Stachybotrys chartarum, Aspergillus versicolor, Fusarium mycotoxin, Saccharomyces cerevisiae* and *Aspergillus flavus* – also have the potential to damage DNA [24-27]. However, in a study of *Paracoccidioides brasiliensis*, this fungus was not able to induce DNA damage [28]. This finding is interesting since *P. brasiliensis* and *L. loboi* present many similarities. The phylogenetic analysis of *L. loboi* presented by Herr *et al.* [29] suggested that *L. loboi* is a dimorphic fungus that is taxonomically a sister species of *P. brasiliensis*. Both belong to the *Onygenales* order along with *Histoplasma capsulatum* and *Blastomyces dermatitides*. In addition *L. loboi* was previously classified as *Paracoccidioides loboi* by Almeida & Lacaz [30].

A similar study on the association between infection, malnutrition and genotoxicity by Ribeiro *et al.* [19] evaluated the genotoxic potential of toxoplasmosis (caused by *T. gondii*) in isogenic mice that were kept on a normal diet or under dietary restriction, and were treated with sulfonamide. The results indicated that dietary restriction and *T. gondii* were able to induce DNA damage in peripheral blood cells, as detected by the comet assay.

There are only two studies relating cancer to Jorge Lobo’s disease. One of them, reported by Baruzzi *et al.* [31] described two cases in Cayabi Indians, who developed cauliflower-like tumors in old lacaziosis scar lesions. The diagnosis of squamous-cell carcinoma was confirmed histologically. In both cases, the tumor was surgically removed, but several months later tumors recurred in both patients.

In another study reported by Nogueira *et al.* [32], a 87-year-old man presented a 30-year history of disseminated cutaneous lesions. He also noted that a lesion on the lower right limb had ulcerated during the prior seven months. Histopathological examination of the ulcerated lesion revealed epithelial tumor islands of varying size in the dermis, and showed the typical yeasts of lacaziosis within the tumor tissue. Based on clinical and histopathological findings, a diagnosis of squamous cell carcinoma in association with lacaziosis was given.

It is important to emphasize that Jorge Lobo’s disease primarily affects rural workers who live in constant contact with the soil and plants, as is the case with the rubber tappers of the Amazon region, and the lesions often affect the lower and upper limbs [1,2]. Thus, the association between the chronic lesions caused by *L. loboi* and overexposure to the sun’s ultraviolet radiation may promote the development of squamous cell carcinoma.

Our results reveal that there is an interaction between lacaziosis, malnutrition and genotoxicity. The malnourished groups and the infected nourished animals showed greater damage compared with healthy animals. The malnourished animals independent of the infectious process showed less damage compared to the infected nourished animals and that there was no difference between the malnourished groups themselves. This important finding suggests that malnutrition in Jorge Lobo’s disease does not exert influence on body weight whereas the genotoxic effect of infection is less in animals under dietary restriction than in those on a normal diet. It has been suggested that malnutrition or lack of some nutrient in the daily diet may interfere with the development or virulence of the fungus, which could influence its relationship with the host. As to this matter, a previous study has found fewer fungi and lower rates of fungal viability in malnourished infected animals.

It should be highlighted that the DNA damage was assessed via the comet assay with peripheral blood instead of skin cells, which are the target cells for development of squamous cell carcinoma. Therefore, the results presented herein may not fully reflect the events that are happening in the skin. A more accurate assessment regarding genotoxicity would require the use of other methodologies.

## Conclusions

In summary, our results indicate that dietary restriction and infection with *L. loboi* may increase DNA damage levels in mice.

### Ethics committee approval

The present study was approved by the Ethics Committee on Animal Experimentation of Botucatu Medical School, UNESP, under protocol no. CEEA 857-2011.
